# Supplementary material for: An Exploratory Study of the Purchase and Consumption of Beef: Geographical and Cultural Differences between Spain and Brazil
Source: Foods. 2022 Jan 5;11(1):129. doi: 10.3390/foods11010129 (PMC8750545; doi:10.3390/foods11010129)
Supplement: Supplementary file 1 [file foods-11-00129-s001.zip › foods-1489558-supplementary.pdf]

Table S1. Beef purchase and consumption habits in Spain and Brazil: Themes and Codes found from the interpretation of consumer behavior through focus groups.

**Section 1 - Satisfaction in consuming beef**
A. "For me, beef is the tastiest, the richest meat." (SP, FG1, F, 18)<br>B. "I like beef because it is very rich and have a good combination with other meals." (SP, FG1, M, 20)<br>C. "I consume beef because of taste, easy access, and habit. I use the other meats to diversify my diet, I would not bear to eat chicken meat every day." (BR, FG2, M, 63)<br>D. "I like the taste of beef, it is juicy… beef is the main meat used in my barbecues." (BR, FG2, F, 35)<br>E. "I take beef twice a day and I would not bear to eat another meat in the same amount that I eat beef." (BR, FG4, M, 27)<br>F. "... if it is for practicality, I prefer chicken meat, for taste I prefer beef." (BR, FG3, M, 25)<br>G. "Beef has more flavor than other meats. In my house the preference was always beef, my father does not even eat any other type of meat." (BR, FG3, F, 24)
**Section 2 - Benefits of consuming beef for health**
A. "What makes me to choose beef is the nutritional contribution, mainly vitamin B12." (SP, FG1, F, 37)<br>B. "Besides that I like it, I take it for the iron intake that they say is not in other meats ... because the nutritional part." (SP, FG1, F, 22)<br>C. "Doctors mention that red meat is bad because of the amount of fat, but it is a good source of iron." (BR, FG4, M, 30)<br>D. "... beef is healthy and my health checks are better every day ..." (SP, FG1, M, 66)<br>E. "I think about nutrients; I prefer to feed my daughter with beef." (BR, FG4, F, 25)<br>F. "I first check that beef does not have fat, because I live with my parents and I have to take care of their diet, but I also believe that it is the meat that provides the most nutrients." (BR, FG5, M, 38)<br>G. "… I have a habit of eating red meat to supplement the protein diet." (BR, FG2, F, 25)
**Section 3 - Reasons for beef consumption due to its convenience**
A. "I like beef because of the types of dishes that can be made." (SP, FG1, F, 25a)<br>B. "… my first option is beef, and, in addition, there are many options of cuts that make a variety of culinary forms." (BR, FG2, M, 26)<br>C. "I agree with the convenience and taste, in addition to beef there are many more options when cooking." (BR, FG3, M, 30)<br>D. "I buy beef because it is tasty, I eat more beef than other meats because it is easy to prepare." (BR, FG5, F, 39)<br>E. "I buy beef for cultural reasons, for easy access, more availability of pieces and at different prices, it can be bought with more or less fat ... I think there are many reasons why I prefer beef." (BR, FG4, M, 24)<br>F. "Beef meat, different from the others, there is more diversity of choice of pieces and different ways of cooking. Most of the product is supplied *in natura*, different from chicken and fish that is sold more frozen. I think meat enriches the diet, it is a very important food source." (BR, FG4, M, 19)<br>G. "I like the taste and also the fat, Brazil is a great producer of beef and we have easy access, it is easier to store… freeze, it has a longer expiration date than the other meats." (BR, FG2, F, 33)<br>H. "I eat and I like beef because of the variety of cuts it has and that can be eaten undercooked, because the other meats cannot do the same." (BR, FG5, M, 24)
**Section 4 - Recognition of beef consumption as a circumstance of Tradition/Custom**

A. "... for the taste, for the tradition passed by the family and for having easy access to it." (BR, FG5, M, 21)
B. “I eat because of tradition; my family eats more beef than other types of meat. I like to vary the diet with fish, but it faces several difficulties, because it is expensive and difficult to find fresh.” (BR, FG5, M, 25)
C. “… ease of access and the cultural issue, which come from generations, we already have the habit of taking beef.” (BR, FG2, M, 27)
D. “I buy because of the taste and the tradition, even more than in my region a lot of barbecue is made. And in my case I usually buy more beef, then chicken or pork.” (BR, FG5, F, 25)
E. “I eat beef because of the taste, my family has a lot of habit of eating beef, it has been passed from generation to generation.” (BR, FG3, F, 27)
F. “In my house we raise cattle, so I always take beef and I like it, by habit.” (SP, FG1, F, 25b)
G. "... it tasty, I always prefer beef, I can't stand eating another type of meat two days in a row, but beef I can do it, because I have a tradition of eating, I like it." (BR, FG2, F, 54)

Section 5 - Characterization of the most important attributes at the time of purchase of beef

A. “About beef, what I first set is the color, then, less fat and then no nerves and cartilage. The redder the steak, the better.” (SP, FG1, M, 20)
B. “That it is fresh is the most important thing, but that it has a greater amount of fat, I also like it, a beautiful marbled, I mean. I also look at the color, texture, brightness in the cut.” (SP, FG1, M, 38)
C. “I look at the color and low fat. I look a lot at the color, it has to look fresh to me, if it's with brown parts I don't buy it. Also the price.” (SP, FG1, F, 19b)
D. “I see if the meat seems fresh to me, and the expiration date. In my house we do look at the pieces.” (SP, FG1, F, 25a)
E. “What we appreciate most when buying beef is that the meat is fresh and deep red, we don't like to freeze and thaw.” (BR, FG5, M, 24)
F. "On the label I look at the price, I like beef with fat, not much, but a little." (BR, FG2, M, 63)
G. “The color of the meat is the most important attribute, it cannot be pink: meat that lost a lot of blood or very dark: old or animal meat that took a hit; and the smell. In my house during the week you eat meat with less fat, such as “alcatra”, which is soft, but has no fat and has no nerves, but for barbecue, the preference is for a little fat: not yellow dark, it means old animal.” (BR, FG2, M, 35)
H. “Color and some fat, that is, marbled or external. I buy meat on a tray sometimes, but I think it loses a lot of liquid. On the label I look at the price and the expiration date, and what really interests me is the piece and the general appearance of it.” (BR, FG2, F, 23)
I. “Color, freshness, the price and the piece I seek more easily, that is, that the meat is clean: without nerves, tendons, etc.” (BR, FG2, F, 54)
J. “I look at the price and expiration date. I think that Brazilians are very disinterested in relation to meat, as the quality is not the worst and we do not have many health problems, we are not interested in knowing other information.” (BR, FG4, F, 37)
K. “I always choose beef it a little fat, I also look at the color, the consistency of the meat, the price and the expiration date.” (BR, FG5, M, 24)
L. “My preference at the time of purchase is for the color, it has to be red, fresh, healthy. I don't understand practically nothing about cutting.” (BR, FG5, F, 23)
M. “I look at the appearance of beef, I choose the meat with a little fat, most of the time I only buy tray meat, because I don't want to stay in the queue, and on the label I look at the expiration date.” (BR, FG4, F, 26)
N. “Color is the most important attribute for me, then I look at fat, for less fat every day, but for a barbecue I like beef with some fat. I almost never buy meat in a vacuum packed, it is once or another.” (BR, FG3, F, 27)

O. “When the meat is dark, I may not have any problem that it is so, but I don't like it anyway, it has to be very red.” (BR, FG5, F, 26)
P. “I check the external quality of the piece. I buy in the supermarket butcher shop, and I don't like vacuum-packed meat, I don't buy it in any way.” (BR, FG3, F, 28a)
Q. “I like meat always with marbled fat, the meat that is going to be cut at the time has to be very red, but the vacuum meat I know is going to be darker, and I don't care.” (BR, FG5, F, 32)
R. "Vacuum-packed meat and matured meat I only buy it when I do barbecue, because it has a differentiated flavor (taste and tenderness), but in normal days, I buy in the butcher shop of the supermarket or traditional one." (BR, FG2, M, 35)
S. “I always look at the fat, the outer layer and if it is marbled, because I think that the fat makes the meat tastier.” (BR, FG3, M, 26)
T. “If it is for the barbecue I prefer with more fat, but for the day to day I prefer that it be with less amount of fat.” (BR, FG4, F, 20a)
U. “For normal meat on weekdays I go to the butcher shop of the supermarket, in promotions even, for weekends or for more special dishes, I go to places that offer a higher quality, with different cuts, etc., even payment more expensive. Color and marbling are always important for me.” (BR, FG4, M, 30)
V. “We pay dearly for a piece of first-class beef without knowing if it will be tender, for example; we have no guarantee.” (BR, FG5, M, 34)
W. “I prefer beef that is from my country, if it is from the same autonomous region better.” (SP, FG1, F, 25a)
X. “Brazil, being a country of continental dimensions, the region of production is sometimes located very far from the region of sacrifice, the animal stresses during the journey and produces a food of worse quality.” (BR, FG2, M, 63)

Section 6 - Influence of the price at the time of purchase of beef

A. “I always look at the price of a kilo of beef. I always buy in the supermarket, in butchery or linear, whatever is cheaper, but I have the feeling that the traditional butcher shop is more expensive.” (SP, FG1, M, 20)
B. “If the meat were cheaper I would consume more, but as it is not, for financial reasons I eat less beef than I would like.” (SP, FG1, F, 37)
C. “I look at the weight/price.” (SP, FG1, F, 25b)
D. “The price is what most influences me at the time of purchase. If the butcher doesn't have what I want, sometimes, I ask what a substitute would be with more or less the same price, outside of that I don't ask the butcher anything else.” (BR, FG3, M, 30)
E. “I buy according to the quality/price ratio.” (BR, FG3, F, 23)
F. “The first thing I look at in beef is the price, if it is very expensive I don’t buy, even knowing that it is a quality cut.” (BR, FG5, F, 20)
G. “I think that, due to the price, an alternative would be to reduce consumption a little, but look for quality meat.” (SP, FG1, F, 25a)
H. “In the years that I have lived alone, I have changed the beef for chicken, but now that I live with my partner I have returned to eat more beef. But before it was about money, not because I didn't like beef.” (SP, FG1, M, 30b)
I. “In my parents' house I eat a lot of beef, but here, I live alone and as a student I do not have much money to consume beef which is an expensive product, but I do so, sometimes I buy.” (SP, FG1, M, 19b)

Section 7 - Importance of the type and location of butchers as a purchasing factor

A. “In the village I buy in the traditional butcher shop, but most of the time I buy in the supermarket because most of the time I have to buy other things and to save time I do everything in the same place.” (SP, FG1, M, 30a)
B. “I buy in the supermarket butcher shop for daily consumption and for barbecue in the traditional butcher shop.” (BR, FG4, M, 37)

C. “In my house if it is in Zaragoza we go to the supermarket, on a tray, and if it is in the village we go to traditional butcher shop. I go to the supermarket that is closer to home.” (SP, FG1, F, 19a)
D. “In my family beef is bought in the supermarket, and for a special meal in the traditional butcher shop. I buy the meat in the supermarket on a tray.” (SP, FG1, F, 18)
E. “My mother buys beef at home and I buy it when I am alone, my mother always buys it at the butcher shop and I buy it at the supermarket on a tray.” (SP, FG1, F, 25a)
F. “In very few cases I go to the butcher shop to buy something, but on a day-to-day basis, they are on trays in the supermarket. I value the price, which part of the animal is better for what I want to prepare.” (SP, FG1, F, 37)
G. “What I look for in an establishment is the nearness, the places I usually buy are all clean and I buy in a tray that in the end what makes the difference is the distance.” (SP, FG1, M, 30b)
H. “I buy at the traditional butcher, because I think that the supermarket's butcher shop doesn't have the same quality. In my house, for the week we look for pieces that are easy to prepare, and on the weekend a somewhat special for barbecue.” (BR, FG4, M, 19)
I. “I have a habit of buying meat at the butcher shop, at the cut, I still can't trust the meat in a vacuum or on a tray.” (BR, FG4, F, 25)
J. “There is a specialized butcher shop near my house, of very good quality, but the meat is more expensive.” (BR, FG3, M, 26)
K. “I buy in the supermarket butcher shop and I think that the quality of the meat depends on the place where the purchase is made, there are butchers in which I know that the quality is better than in others. The only information I ask for the butcher is to see the piece I am going to buy, I do not ask what piece I should buy, I also like meat with fat. The establishment cannot have a bad smell or mosquitoes.” (BR, FG3, F, 36)
L. “I buy in the supermarket that I have confidence, that it is clean, that it has no smell. And the distance influence, I prefer establishments near my house.” (BR, FG3, M, 32)
M. “I buy at the supermarket butcher shop. The place of purchase must have hygiene, all clean and organized.” (BR, FG3, F, 22)
N. “I buy meat in the butcher shop of the supermarket and I chose the place for the quality, I bought two, three times there and the meat was good, so I prioritize the same establishment.” (BR, FG5, M, 34)
O. “I have the tradition of always buying from the same butcher, because from the first time I went there, I saw that the cleanliness and credibility of the establishment and the butcher are good and I trust the product there.” (BR, FG5, M, 24)
P. “The hygiene of the location is the most important. When I moved to that city, I looked for butchers that I thought were good, and many times I asked him the origin, but in the end, when I had already chosen the places that I liked and did not ask again about the origin.” (BR, FG5, F, 32)
Q. “For me it is indifferent if it is on a tray, empty or fresh, the important thing is the place where it was bought, to know if the product is reliable, if the meat is in good condition.” (BR, FG4, F, 31)
R. “I ask the butcher to clean and fillet the meat and to choose a piece with some fat, but I don't ask for any information about the piece name or how to make it, for example.” (BR, FG4, M, 21)
S. “When I do barbecue I ask what the butcher offers me, not because I don't know the name of the piece, but because I want what is best at the time of purchase.” (BR, FG5, M, 27)
T. “I trust that the butcher tells me that it is good, and I also look at it, but sometimes I am mistaken, because the beef is difficult to know by its appearance if it will turn out well or not. When I bought in traditional butcher shop I was more satisfied than in the supermarket, the advantage of knowing the butcher, reserves the best meats.” (SP, FG1, F, 70)
U. “I live alone and buy meat in the supermarket on tray, or vacuum packed to make barbecue, which seems easier to me and the opinion of the butcher does not matter at the time of purchase.” (BR, FG2, M, 21)

Section 8 - Use the information on the label and type of packaging

A. “On the label I only look at the price, I decide what I'm going to buy, but I always buy the cheapest one.” (SP, FG1, M, 19b)

B. “On the label I look at the expiration date, the processing date, the price, if the filter is full of exudate or not. Here most of the trays are packed in the plastic film, and do not have a very large expiration date time.” (BR, FG5, F, 32)

C. “On the label I look at the expiration date and I also like products that are produced in the area.” (SP, FG1, F, 18)

D. "I do not look at the cuts, I look at expiration date, price, kilo." (SP, FG1, M, 30)

E. “On the label I look at the expiration date, price, appearance of the meat, origin, the packaging cannot have lost its vacuum.” (BR, FG2, M, 35)

F. “The recommendation on “how to cook” on the label would be useful for me. It would be good to have information on how to feed and raise animals. I don't know how to differentiate the races, but if it is described on the label over time I will know how to differentiate it.” (BR, FG2, M, 21)

G. "On the label I only see the date it was packed, because I think it is the only security I have, I currently buy a lot of tray meat, which comes cleaner and cut thinner than when ordered directly at the butcher shop." (BR, FG3, F, 36)

H. “The first thing I see on the label is the price per kilo, I have never looked at the expiration date of the meat, I look at other products, but not the meat. I also buy a larger quantity and freeze it.” (BR, FG3, F, 32)

I. “I believe that the important information that comes on the beef label is expiration date. We are moved to good experiences, even if the person has no knowledge about the characteristics of the product, if he buys one with a specific characteristic and likes it, he will repeat the purchase.” (BR, FG3, M, 26)

J. “New cuts are currently coming out, and people don't know very well what to do with them, and it would be valid to have information on how to prepare the product, because it is not known from which part of the animal that piece came out.” (BR, FG3, M, 36)

K. “I think that the information on race, age, way of raising animals is for a niche of more differentiated people, with more knowledge about beef, not the general population.” (BR, FG3, M, 25)

L. “I look in the tray if it is well stored, if the packet is intact, and in the quantity I want.” (BR, FG5, F, 23)

M. “I only look at the weight and the price, but I avoid buying on the tray, only in the latter case because I think it was very manipulated, it seems that it is not as fresh as the one cut at the moment.” (BR, FG5, M, 25)

N. “I am very influenced by color, and I think that more information on the label is valid, but in my case I am not interested, I already have my preferences. I look at the price, and since I do not buy tray meat or vacuum-packed, I am not interested in the label, I do not see how it would be possible to know about this information in a butcher shop.” (BR, FG3, M, 32)

O. “I look at the price, and the appearance of the meat itself, like at the color, but nothing of the specific label, or date of packaging, expiration date or where it comes from. I would pay more for softness and taste, but not for handling, well-being, or traceability.” (BR, FG3, F, 22)

P. “I believe that vacuum-packed beef is much more expensive than cut meat, but it is more tender and I think the quality is higher.” (BR, FG2, F, 23)

Q. “I do not buy meat vacuum-packed, I think it has no quality, I do not like the smell. I don't like to buy on the tray because the top steak is red and the bottom steak is always dark. Color and smell are the most important aspects.” (BR, FG4, F, 37)

R. “The only information I consider important is the slaughter and the race, there is beef industry that labels the animal as an "early cattle" that catches my attention. I think it will make a difference in meat quality.” (BR, FG3, F, 23)

S. “It should be taken into account that the niche in Brazil is discriminatory, for example, if it has an older animal tag, the population will find that it is an old animal and that the meat is hard, etc. I think that the Brazilian population is not prepared for animal information on the label.” (BR, FG4, M, 37)
T. “I think that stratifying the animals by category means that they have demand for one or another category of animal, because there will be a difference between the purchase preference of younger animals and leftover older animals, the latter will be acquired by classes favored because they can have one more price in mind.” (BR, FG4, M, 30)

Section 9 - Ideologies regarding the certification of beef products

A. “I am interested in knowing the information about the animal's life, where it was raised, etc. Because here in Brazil as we have very large animal breeding, I am afraid of how these animals are created, many atrocities and mistreatment can occur. So, if I had stores to buy, and it wasn't much more expensive than normal, I would buy meat that was organic, or that had a guarantee of well-being and other types of certification. Pasture fed animals are healthier.” (BR, FG3, F, 24)
B. “Regarding the environment where the animal was raised; in eggs, for example, it is very easy to observe the information of whether it is ecological, animals raised in freedom, etc., but in beef I have never seen this information. And I am interested in this information, because for the eggs I am interested, for the beef it would be the same… animal ethics interests me.” (SP, FG1, M, 30)
C. “Information on how animals are treated, well-being, has not yet influenced my consumption, but it is an issue that I think affects many people, causing them to stop eating meat.” (BR, FG5, M, 23)
D. “I come from a region of very strong livestock production and I know the cuts and how to make them, I buy in butchers, but I look for them to be good establishments, that I know where the meat comes, a place with good hygiene, or meat that is certified.” (BR, FG2, M, 63)
E. “I like to buy organic chicken meat because I think that chicken is an animal that gains weight very fast and may not have a good quality, but beef doesn't worry me.” (BR, FG5, F, 21)
F. "I think the younger generation is more concerned with the origin of the animal and how it was raised by the amount of meat they consume." (BR, FG2, F, 33)
G. "Race is important because I think they would have people who would pay more for it, because some races are more famous for their quality than others." (BR, FG5, M, 25)
H. “I sometimes notice and like, is the origin, that the meat is from the Pyrenees, for example, native races, that I value it.” (SP, FG1, F, 25a)
I. “I would pay more for a beef with a quality seal for knowing that meat is healthier.” (BR, FG2, F, 19)
J. “If there was a meat with a quality seal I would prefer to buy the one that had a certification, but the price would be the most important factor for my choice.” (BR, FG2, F, 25)
K. “It seems to me that in Brazil, the more companies created for the control of the processes, the more I have doubts if they are correct, the information goes through the hands of many people and I do not trust them to be suitable, for me the most of them are corrupt. The laws ask for things that are not feasible for some producers to do, and that causes fraud.” (BR, FG5, M, 29)
L. “I have difficulty knowing if the product is really what is being promised, if it is truly organic, for example. ... I don't trust in guarantee certifications.” (BR, FG3, M, 24)
M. "I don’t purchase a product with a certificate, we will pay more for that type of product and are not sure if the certification is true." (BR, FG5, F, 26)
N. “I think the more information the better, and they would be quite useful, even though I wouldn't pay more for them. I trust the information on the labels of recognized companies, of meat boutiques, for example, because they will not want to damage their sales with products that are reduced quality.” (BR, FG3, F, 27)
O. “I would only be willing to pay more for attributes that improve the quality of beef, that I perceived the difference when consuming, otherwise I don't care about any other issue. In

general, I think that Brazilians want a quality product, but they don't look for information, and they don't want to pay more for it." (BR, FG5, M, 21)
P. "If the consumer demands a lot from the markets, they will not be able to pay for all the information that they have questioned." (BR, FG5, M, 27)

Section 10 - Usefulness of traceability information

A. "I know what traceability is, but I don't know on the label, where I have to look at, I don't know if they put it either." (SP, FG1, M, 19b)
B. "I have no idea what to do with traceability information, I have no interest in that information, although they may somehow be important." (BR, FG3, F, 36)
C. "I think we are very concerned about the characteristics we can see of the product, and not with those that we cannot, such as traceability." (BR, FG3, F, 22)
D. "I have never seen a label with traceability information. For a barcode or a QR code, nobody is going to look for the information, it is very complicated to do all this." (BR, FG3, F, 25)
E. "We do not look at the expiration date of the product, which can cause small problems such as some food infections, imagine if we are going to worry about the origin of the beef, at least I have never worried." (BR, FG3, F, 28b)
F. "I don't buy tray meat, I don't know about traceability information and I've never worried about knowing it." (SP, FG1, F, 70)
G. "I think traceability is what people least notice about the label. In addition, traceability information in traditional butcher shops is almost impossible to use, nobody asks where the label is regarding the piece of meat when buying." (SP, FG1, M, 30a)
H. "Traceability information on the label is never a purchase condition in my case, I see it as less useful information for the final consumer and more for companies." (SP, FG1, M, 30b)
I. "I think that the traceability information should be clearer, I see the information from the slaughterhouse, what do I do, do I go to the slaughterhouse to confer? I just want to know if the product is from my region, or from Spain, because I don't want a product that has traveled half the world, that loses its quality. Traceability is like any car insurance, for example, it must be taken and I hope it is not necessary to use it." (SP, FG1, M, 38)
J. "I would not use any machine, nor the mobile or other methods to read the traceability information of the product, the best would be a certification seal, because with the seal all the proposed parameters are filled." (BR, FG4, M, 37)
K. "I think that traceability information is important to the butcher, for the consumer it is not. The traceability information available through a number will not get anyone's attention." (BR, FG3, F, 26)
L. "After the propaganda of a famous beef brand in Brazil, consumers' vision of the concept of traceability improved considerably. I buy meat with traceability information, but that is not the reason for my purchase. I buy because of the appearance of the beef and I didn't feel more confident about the meat being traced." (BR, FG4, F, 23)
M. "Traceability should be in a very accessible and concrete way, just to ensure that this product is controlled, and for possible consultations have a minimum of information." (BR, FG5, M, 34)

Section 11 - Credibility of traceability information

N. "I would only believe in traceability information if there was a more effective control, which I currently do not believe." (BR, FG5, F, 32)
O. "I don't trust traceability information for corruption in the agricultural sector." (BR, FG5, F, 29)
P. "Our cultural problems, of corrupt and dishonest people, make the objectives, such as that of correctly tracing animals, out of our reality, I am afraid that such information is falsified." (BR, FG2, M, 35)
Q. "If traceability were reliable, I would be willing to pay more for meat with that information." (BR, FG2, M, 26b)

R. “I tried to read the QR code information of a piece of meat from a famous supermarket and I did not get it, the meat code was not available. I am afraid of corruption in the area of certification.” (BR, FG4, M, 27)
S. “I trust the brand mark more than a traceability certification. If I knew of an outbreak of diseases in a certain place, I would see if the meat I am buying does not come from there, but the information had to be accessible, so I would simply stop buying beef in that period of outbreak. Even if I have traceability, I think there may be failures in this process.” (BR, FG2, F, 24)
T. “Knowing the origin of the animal would be important, I give preference to meat that comes from a production closer to where I am, not for fear of disease, but for financially helping my environment. I trust the traceability process of large companies.” (BR, FG2, F, 33)

Section 12 - Reasoning on traceability directions

A. “I believe, if one day we go through a serious food crisis, consumer thinking can change. I also believe that the responsible for informing people of the attributes of the meat is the butcher.” (BR, FG4, F, 20b)
B. "Regarding the traceability of meat, until nothing serious happens, we will not be aware that it is important or that it is there." (SP, FG1, F, 22)
C. "I would only use traceability information, if we went through a food crisis." (BR, FG5, M, 27)
D. “In Brazil we don't have many problems with health, which makes consumers and producers a little more relaxed, and we don't worry, because we didn't have serious cases of illnesses.” (BR, FG2, M, 63)
E. “In Europe the countries are very small and easy to control, here [Brazil] we are a continental country and it is not easy to implement a unique traceability system. I think you should have basic information about the production system and the animal on the label, but that it is easily accessible. The information of my interest is quality and not food safety.” (BR, FG3, M, 35)
F. “I don't know if in Brazil we have a satisfactory control system to perform all the processes well.” (BR, FG3, M, 23)
G. “I think that over time people will learn to manage traceability information, the problem is that we have almost no access to it. The Brazilian is very confident in the brand of the product, if it is known it is trustworthy, but in reality that is not a guarantee, because we have no information beyond the brand's fame.” (BR, FG3, F, 21)
H. “You cannot rob people of knowledge, say that it is not necessary to put the information of the product because it is not interesting, somehow you have to teach consumers to give value to be information and expect people to adapt.” (BR, FG4, M, 25)

* Note - Coding of the profiles of the participants of the group discussion. Country: (SP - Spain, BR - Brazil); Discussion Group: (FG1 = Zaragoza, FG2 = Minas Gerais, FG3 = Sao Paulo, FG4 = Parana, FG5 = Santa Catarina); Gender: (M = male, F = female); Age: (in years).
